# Multiple CRF01_AE/CRF07_BC Recombinants Enhanced the HIV-1 Epidemic Complexity Among MSM in Shenyang City, Northeast China

**DOI:** 10.3389/fmicb.2022.855049

**Published:** 2022-05-12

**Authors:** Shan He, Wei Song, Gang Guo, Qiang Li, Minghui An, Bin Zhao, Yang Gao, Wen Tian, Lin Wang, Hong Shang, Xiaoxu Han

**Affiliations:** ^1^NHC Key Laboratory of AIDS Immunology (China Medical University), National Clinical Research Center for Laboratory Medicine, The First Affiliated Hospital of China Medical University, Shenyang, China; ^2^Chinese Academy of Medical Sciences Research Unit (No. 2019RU017), China Medical University, Shenyang, China; ^3^Key Laboratory of AIDS Immunology of Liaoning Province, Shenyang, China; ^4^Department of Food Safety and Nutrition, Shenyang Center for Health Service and Administrative Law Enforcement (Shenyang Center for Disease Control and Prevention), Shenyang, China; ^5^Department of Clinical Laboratory, The Sixth People’s Hospital of Shenyang, Shenyang, China

**Keywords:** HIV-1, men who have sex with men, unique recombinant forms, transmission, phylogenetics

## Abstract

The transmission of Unique Recombinant Forms (URFs) has complicated the molecular epidemic of HIV-1. This increasing genetic diversity has implications for prevention surveillance, diagnosis, and vaccine design. In this study, we characterized the HIV-1 URFs from 135 newly diagnosed HIV-1 infected cases between 2016 and 2020 in Shenyang, northeast China and analyzed the evolutionary relationship of them by phylogenetic and recombination approaches. Among 135 URFs, we found that the CRF01_AE/CRF07_BC recombinants were the most common (81.5%, 110/135), followed by CRF01_AE/B (11.9%, 16/135), B/C (3.7%, 5/135), and others (3.0%, 4/135). 94.8% (128/135) of patients infected by URFs were through homosexual contact. Among 110 URFs_0107, 60 (54.5%) formed 11 subclusters (branch support value = 1) and shared the consistent recombination structure, respectively. Four subclusters have caused small-scale spread among different high-risk populations. Although the recombination structures of URFs_0107 are various, the hotspots of recombinants gathered between position 2,508 and 2,627 (relative to the HXB2 position). Moreover, the CRF07_BC and CRF01AE fragments of URFs_0107 were mainly derived from the MSM population. In brief, our results reveal the complex recombinant modes and the high transmission risk of URFs_0107, which calls for more attention on the new URFs_0107 monitoring and strict control in the areas led by homosexual transmission route.

## Introduction

The occurrence of human immunodeficiency virus type 1 (HIV-1) recombination significantly increases the genetic complexity and enhances viral evolution and adaptation (Zhang et al., 2010). By far, more than 118 circulating recombinant forms (CRFs) and increasing unique recombinant forms (URFs), the latter was isolated only from single individuals (Hemelaar, 2013), have been reported in the Los Alamos National Laboratory HIV database.^1^ A systematic literature search and global survey study on HIV-1 diversity from 1990 to 2015 demonstrated the proportion of HIV-1 infections due to recombinants is highest in Southeast Asia, followed in China, and West and Central Africa, mainly by CRF01_AE and CRF02_AG, respectively (Hemelaar et al., 2020b). Meanwhile, URFs have also contributed greatly to the HIV-1 epidemic in Central, West, and East Africa, as well as Latin America (Hemelaar et al., 2020a), and led to up to 30% of infections in regions where multiple subtypes coexist (Hemelaar et al., 2020b). Therefore, the genetic diversity of HIV-1 has posed huge challenges to prevention, surveillance, drug resistance, treatments, and the broad vaccines development (Nájera et al., 2002; Nora et al., 2007).

China is one of the countries with the most subtypes and complexity of HIV-1 epidemic in the world (Hemelaar et al., 2020b). The co-circulation of various subtypes of strains and the occurrence of multiple infections have resulted in the continuous emergence of new recombinants (Vuilleumier and Bonhoeffer, 2015). In the 1990s, the generation of CRF07_BC and CRF08_BC was due to the high incidence of B/C recombination events in Yunnan, the gateway of HIV-1 in China (McClutchan et al., 2002; Feng et al., 2016; Chen et al., 2019), which had a profound impact on the epidemic of HIV-1 (Yuan et al., 2016; Zhang et al., 2017). Another circulating subtype CRF55_01B is composed of CRF01_AE and B strains, which can be traced back to MSM in Shenzhen, Guangdong in the 2000s, and have been currently found throughout the country (Han et al., 2013a; Zai et al., 2020). The latest national HIV molecular epidemiological survey shows that novel CRFs and URFs account for as high as 3.5% and 5.0%, respectively, which basically cover all provinces and cities in China. Among them, recombinants with CRF01_AE and CRF07_BC as parents are the most common, mainly circulating among men who have sex with men (MSM).

The incidence of HIV-1 among MSM in China continues to rise, especially in large cities such as Beijing, Shanghai, Chongqing, Kunming, Shenzhen, and Shenyang (Wu et al., 2013; Qi et al., 2015). Multiple sexual partners and unprotected high-risk behaviors among MSM lead to the persistent emergence of URFs (Xu et al., 2010; Yin et al., 2019). HIV-1 recombination has been reported to enhance biological fitness (Njai et al., 2006), escape the host immune response (Streeck et al., 2008), and generate variants of dual or multi-drug resistant (Moutouh et al., 1996; Nikolaitchik et al., 2015). Although the increasing number of distinct CRFs and URFs sequences is submitted to the HIV-1 database, most of them are used for sequence annotation and reporting of characteristic samples (Hao et al., 2019; Li et al., 2019; Chang et al., 2020; Jiang et al., 2020), and hence may not accurately represent the current epidemic of HIV-1 URFs, which brings difficulties to guide the precise prevention and control.

In the present study, we collected all newly diagnosed infections with URFs from 2016 to 2020 in Shenyang, a transportation hub in northeast China, where HIV-1 transmission is predominantly through the homosexual route, with multiple subtypes co-existing and an increasing proportion of recombinants (Jin-ping et al., 2018; Liu et al., 2020). It aimed to capture the full profile of the ongoing epidemic of URFs in Shenyang through characterizing the phylogeny and recombination patterns, prove critical of tracing the source to cut the chain of transmission, as well as have great significance for public intervention to control the HIV-1 URFs epidemic among Chinese MSM.

## Materials and Methods

### Data Collection and Study Samples

A total of 135 HIV-1 URFs pol sequences (HXB2: 2268-3278) from 2016 to 2020 were screened out by phylogenetic and RIP (recombinant identification program) from a local HIV-1 drug resistance database built by the First Affiliated Hospital of China Medical University (Zhao et al., 2021). The database was established by all 3,934 newly diagnosed HIV-1 infections in Shenyang between 2016 and 2020. Social-demographic information including date of diagnosis, gender, age, ethnic group, marriage status, education, and transmission routes was collected. Plasma samples were collected at the time of diagnosis and stored at −80°C. All participants signed informed consent forms, and the Ethical approval was obtained from the ethics committee of the First Affiliated Hospital of China Medical University.

### Extraction, Amplification, and Sequencing

5′ half-genome (HXB2: 790-5056) amplifications of partial patients were performed. The RNA was transcribed into cDNA using Superscript III Reverse Transcriptase (Life Technologies, Carlsbad, CA, United States), then amplified by nested PCR using Platinum Taq DNA Polymerase High Fidelity (Life Technologies, Carlsbad, CA, United States). The primers of reverse transcription and PCR amplification, reaction system, and condition were reported previously (Zhao et al., 2011; Sanchez et al., 2014). The amplified DNA fragments were purified and sequenced by a commercial company (Huada, Beijing).

### Phylogenetic Analysis

The raw sequences were cleaned and assembled by Sequencer software v.5.4 (Gene Codes, Ann Arbor, MI, United States), and then were compared using the BLAST online tool (see footnote 1) to avoid potential experiment cross-contamination. The final sequences were aligned using HIV Align online tool (see footnote 1) and conducted manually using BioEdit 7.0 (Tippmann, 2004).^2^ Phylogenetic trees of pol region were constructed using the Maximum-Likelihood (ML) method under the General Time Reversible (GTR) + I + G nucleotide substitution model with 1,000 replicates using IQ-Tree v2.0.5 (Nguyen et al., 2015), subsequently visualized by FigTree v1.4.2 (Price et al., 2010). The CRF01_AE, CRF07_BC, CRF_01B, and pure genotypes of M group were used as reference sequences and downloaded from the Los Alamos HIV Database.

### Recombination Analysis

Recombination structures were preliminary analyzed using two online software tools (recombinant identification program and jumping profile Hidden Markov Model; see Footnote 1). Further, the recombination breakpoints were confirmed using SimPlot (Lole et al., 1999; version 3.5.1) with the following parameters: 200 nucleotides (nt) window, 20 nt step size, and 100 bootstrap replicates. Five subtypes were selected as reference sequences in Simplot analyses including CRF01_AE (JX960615), CRF07_BC (JX960601), B (U71182), C (AF067155), and subtype J (AF082395). And the recombination mosaic map was generated using the online Recombinant HIV-1 Drawing Tool.^3^

Recombination fragments (as determined using jpHMM and Simplot bootscanning) were phylogenetically studied using IQ-Tree v2.0.5. For some URFs with more than one recombination breakpoints, the pure and same subtype fragments were generally connected together for analysis to retain more sequence information. The subregion trees were generated by URFs recombination fragments using ML method under (GTR) + I + G model, including seven CRF01_AE lineage in China (Feng et al., 2013), CRF01_AE lineage in Thailand and Africa, as well as pure genotypes of HIV-1 group A as reference sequences.

### The Identification of HIV Infection Status by Limiting-Antigen Avidity Enzyme Immunoassay

Plasma specimens were tested with the Lag-Avidity EIA according to the manufacturer’s instructions (Maxim Biomedical, Rockville, MD, United States). Specimens with initial ODn > 2.0 are considered long-term HIV-1 infection but if ODn ≤ 2.0, the specimens are tested against in triplicate to confirm their ODn values. In confirmatory testing, if the ODn of the specimen is >1.5, the specimen is considered a long-term infection. If ODn >0.4, but ≤1.5, the specimen is considered a recent HIV infection (RHI). The specimens which ODn values lower than 0.4 need further confirm HIV seropositivity *via* Western blot assay.

## Results

### The Recombinant Profile and Social-Demographic Characteristics of 135 URFs Subjects

From 2016 to 2020, a total of 135 cases were identified infection with HIV-1 URFs in Shenyang. Among these cases, diverse recombinant combinations were identified, including the URFs_0107 (81.5%, 110/135), followed by URFs_01B (11.9%, 16/135), URFs_BC (3.7%, 5/135), and other unique recombinants (3.0%, 4/135). Overall, the proportion of URFs_0107 increased from 70.8% (17/24) in 2016 to 88.9% (16/18) in 2020, while the URFs_01B gradually declined from 20.8% (5/24) to 5.6% (1/18; Supplementary Figure S1). The social-demographic characteristics of 135 individuals were summarized in Table 1. Of the 110 identified URFs_0107, the majority of the subjects was male (99.1%, 109/110), younger than 35 years old (77.3%, 85/110), being “single” (77.3%, 85/110), Han ethnicity (84.5%, 93/110), had achieved beyond compulsory education (94.6%, 104/110) and predominantly transmitted by MSM (81.8%, 90/110). All cases of URFs_01B infection were male through homosexual behavior and had similar demographic characteristics with URFs_0107. On the contrary, for URFs_BC and other recombinants, most cases were female and older than 35 years and infected through heterosexual contact.

**Table 1:** Demographic characteristics of 135 HIV-1 recombinants infected patients in this study.

	TOTAL (*n* = 135)	HIV subtype			
		URF_0107 (*n* = 110)	URF_01B (*n* = 16)	URF_BC (*n* = 5)	Other (*n* = 4)
	*N* (%)	*N* (%)	*N* (%)	*N* (%)	*N* (%)
Year					
2016	24 (17.8)	17 (15.5)	5 (31.3)	2 (40.0)	0 (0)
2017	23 (17.0)	21 (19.1)	2 (12.5)	0 (0)	0 (0)
2018	34 (25.2)	26 (23.6)	3 (18.8)	2 (40.0)	3 (75.0)
2019	36 (26.7)	30 (27.3)	5 (31.3)	0 (0)	1 (25.0)
2020	18 (13.3)	16 (14.5)	1 (6.3)	1 (20.0)	0 (0)
Gender					
Male	128 (94.8)	109 (99.1)	16 (100)	2 (40.0)	1 (25.0)
Female	7 (5.2)	1 (0.9)	0 (0)	3 (60.0)	3 (75.0)
Age					
<25	51 (37.8)	42 (38.2)	8 (50.0)	0 (0)	1 (25.0)
25–34	48 (35.6)	43 (39.1)	4 (25.0)	1 (20.0)	0 (0)
35–44	9 (6.7)	6 (5.5)	1 (6.3)	0 (0)	2 (50.0)
≥45	27 (20.0)	19 (17.3)	3 (18.8)	4 (80.0)	1 (25.0)
Ethnic group					
Han	113 (83.7)	93 (84.5)	14 (87.5)	4 (80.0)	2 (50.0)
Minority	22 (16.3)	17 (15.5)	2 (12.5)	1 (20.0)	2 (50.0)
Marriage status					
Single	99 (73.3)	85 (77.3)	12 (75.0)	1 (20.0)	1 (25.0)
Married	14 (10.4)	10 (9.1)	2 (12.5)	1 (20.0)	1 (25.0)
Divorced	21 (15.6)	14 (12.7)	2 (12.5)	3 (60.0)	2 (50.0)
UN	1 (0.7)	1 (0.9)	0 (0)	0 (0)	0 (0)
Education					
Illiterate	3 (2.2)	2 (1.8)	0 (0)	0 (0)	1 (25.0)
Primary education	4 (3.0)	4 (3.6)	0 (0)	0 (0)	0 (0)
Secondary education	30 (22.2)	20 (18.2)	6 (37.5)	3 (60.0)	1 (25.0)
Higher education	98 (72.6)	84 (76.4)	10 (62.5)	2 (40.0)	2 (50.0)
Transmission routes					
MSM	108 (80.0)	90 (81.8)	16 (100)	1 (20.0)	1 (25.0)
Hetero	26 (19.3)	20 (18.2)	0 (0)	4 (80.0)	2 (50.0)
MTC	1 (0.7)	0 (0)	0 (0)	0 (0)	1 (25.0)
LAg-Avidity EIA					
Recent	43 (31.9)	36 (32.7)	5 (31.3)	1 (20.0)	1 (25.0)
LT	68 (50.4)	52 (47.3)	10 (62.5)	3 (60.0)	3 (75.0)
NA	24 (17.8)	22 (20.0)	1 (6.3)	1 (20.0)	0 (0)

MSM, men who have sex with men; Hetero, heterosexuals; MTC, mother-to-child; LAg-Avidity EIA, limiting-antigen avidity enzyme immunoassay; Recent, recent infection; LT, long-term infection; and NA, data not available.

### The Identification of Potential Transmission Clusters of HIV-1 URFs

**Figure 1:**
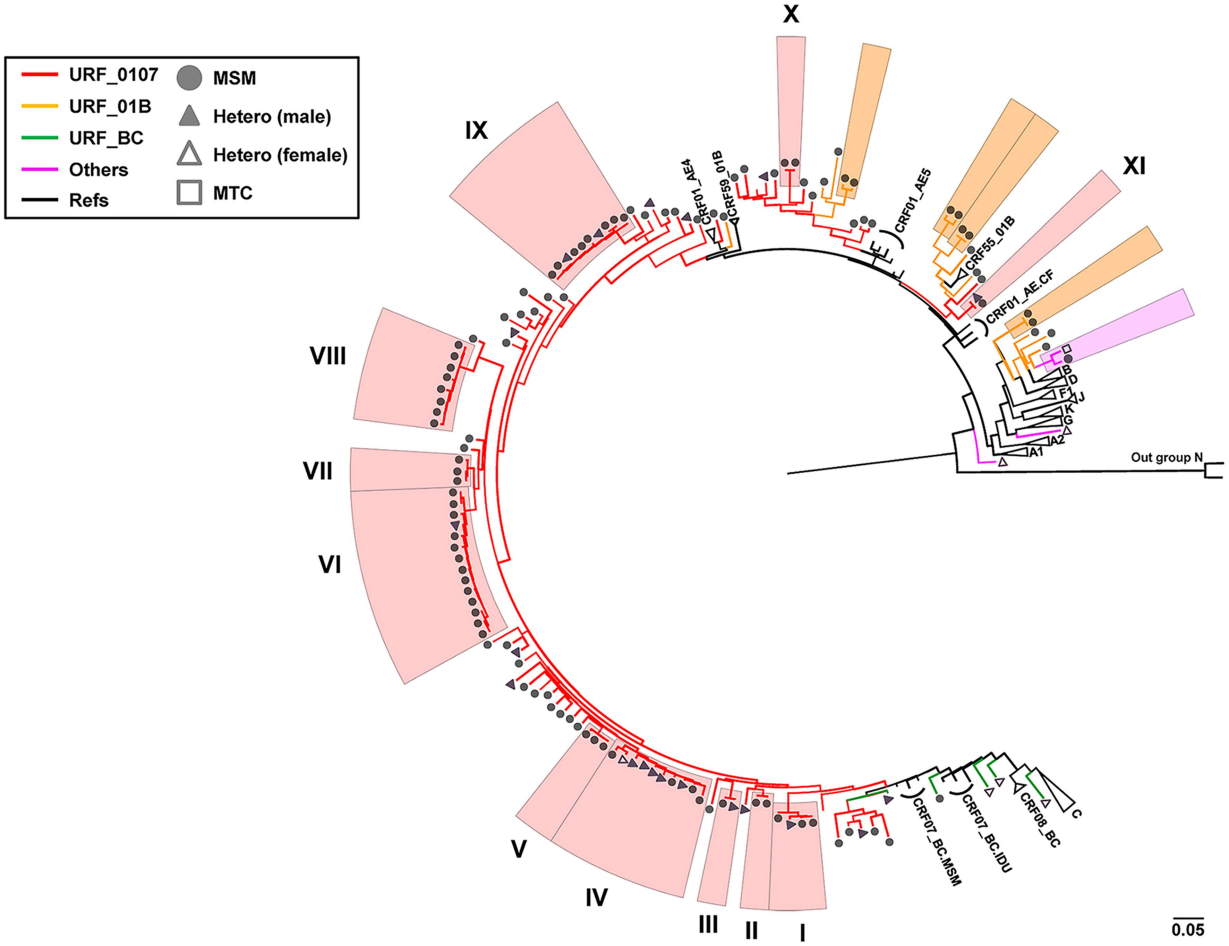
Phylogenetic tree analysis of all 135 HIV-1 URFs in Shenyang during 2016–2020. The topology of tree was constructed using Maximum-likelihood method under GTR + I + G model with 1,000 bootstrap replicates by IQ-TREE. ALL the reference sequences were downloaded from HIV-1 database and using the subtype N as outgroup. Red branches denoted genotypes URFs_0107, orange branches denoted genotypes URFs_01B, green branches denoted genotypes URFs_BC, and pink branches denoted genotypes other recombinants like URFs_BD, URFs_01A1, and URFs_02A1. Clusters with bootstrap value 1 were defined as the potential transmission clusters and indicated by shadows corresponding to the color of the genotypes, respectively. The sample of transmission routes was represented by different symbol (circle, MSM; triangle, Hetero; and square, MTC). The scale length indicated 5% nucleotide sequence divergence.

To explore the phylogenetic relationship between URFs, the ML trees were reconstructed with *pol* sequences (HXB2: 2268-3278bp; Figure 1). Among 135 unique recombinant viruses, 16 potential transmission clusters with bootstrap values 1 and consistent recombination mode were inferred, including 70 subjects with at least two sequences in each cluster. Transmission clusters were composed of URFs_0107 (*n* = 60), URFs_01B (*n* = 8), and URF_BD (*n* = 2), respectively. The remaining 65 recombinants were interspersed widely throughout the phylogenetic tree.

Four larger URFs_0107 transmission clusters (IV, VI, VIII, and IX) were identified, consisting of 8–14 individuals (Table 2). Among them, cluster IV (*n* = 10) was observed between 2016 and 2019, and antibody affinity results suggested that 80% were chronically infected. More than half of infections were spread among older men (age range from 58 to 74) by heterosexual. In contrast, for the remaining three clusters, cluster VI (*n* = 14) and IX (*n* = 10) were diagnosed among the younger males who had been infected through a homosexual (*n* = 21) or heterosexual (*n* = 3) route and cluster VIII (*n* = 8) were transmitted only through homosexual behavior. In addition, more than 50% of individuals of three clusters were recent HIV-1 infections.

**Table 2:** The main epidemiological characteristics of 11 clusters of URFs_0107 strains.

	TOTAL (*n* = 60)	Cluster										
		I (*n* = 4)	II (*n* = 2)	III (*n* = 2)	IV (*n* = 10)	V (*n* = 3)	VI (*n* = 14)	VII (*n* = 3)	VIII(*n* = 8)	IX (*n* = 10)	X (*n* = 2)	XI (*n* = 2)
		*N* (%)	*N* (%)	*N* (%)	*N* (%)	*N* (%)	*N* (%)	*N* (%)	*N* (%)	*N* (%)	*N* (%)	*N* (%)
Gender												
Male	59 (98.3)	4 (100)	2 (100)	2 (100)	9 (90.0)	3 (100)	14 (100)	3 (100)	8 (100)	10 (100)	2 (100)	2 (100)
Female	1 (1.7)	0 (0)	0 (0)	0 (0)	1 (10.0)	0 (0)	0 (0)	0 (0)	0 (0)	0 (0)	0 (0)	0 (0)
Age												
<25	27 (45.0)	1 (25.0)	0 (0)	0 (0)	0 (0)	2 (66.7)	8 (57.1)	2 (66.7)	5 (62.5)	6 (60.0)	2 (100)	1 (50.0)
25–34	18 (30.0)	3 (75.0)	1 (50.0)	2 (100)	0 (0)	0 (0)	3 (21.4)	1 (33.3)	3 (37.5)	4 (40.0)	0 (0)	1 (50.0)
35–44	3 (5.0)	0 (0)	1 (50.0)	0 (0)	0 (0)	0 (0)	2 (14.3)	0 (0)	0 (0)	0 (0)	0 (0)	0 (0)
≥45	12 (20.0)	0 (0)	0 (0)	0 (0)	10 (100)	1 (33.3)	1 (7.1)	0 (0)	0 (0)	0 (0)	0 (0)	0 (0)
Transmission routes												
MSM	48 (80.0)	3 (75.0)	2 (100)	1 (50.0)	4 (40.0)	3 (100)	13 (92.9)	3 (100)	8 (100)	8 (80.0)	2 (100)	1 (50.0)
Hetero	12 (20.0)	1 (25.0)	0 (0)	1 (50.0)	6 (60.0)	0 (0)	1 (7.1)	0 (0)	0 (0)	2 (20.0)	0 (0)	1 (50.0)
Diagnosis year												
2016	9 (15.0)	0 (0)	0 (0)	0 (0)	3 (30.0)	0 (0)	0 (0)	0 (0)	4 (50.0)	1 (10.0)	0 (0)	1 (50.0)
2017	9 (15.0)	1 (25.0)	0 (0)	0 (0)	1 (10.0)	0 (0)	5 (35.7)	0 (0)	2 (25.0)	0 (0)	0 (0)	0 (0)
2018	18 (30.0)	1 (25.0)	2 (100)	2 (100)	3 (30.0)	1 (33.3)	3 (21.4)	2 (66.7)	1 (12.5)	3 (30.0)	0 (0)	0 (0)
2019	18 (30.0)	2 (50.0)	0 (0)	0 (0)	3 (30.0)	2 (66.7)	3 (21.4)	1 (33.3)	1 (12.5)	5 (50.0)	0 (0)	1 (50.0)
2020	6 (10.0)	0 (0)	0 (0)	0 (0)	0 (0)	0 (0)	3 (21.4)	0 (0)	0 (0)	1 (10.0)	2 (100)	0 (0)
LAg-Avidity EIA												
Recent	27 (45.0)	4 (100)	1 (50.0)	0 (0)	0 (0)	3 (100)	7 (50.0)	2 (66.7)	5 (62.5)	5 (50.0)	0 (0)	0 (0)
LT	23 (38.3)	0 (0)	1 (50.0)	2 (100)	8 (80.0)	0 (0)	4 (28.6)	1 (33.3)	2 (25.0)	3 (30.0)	0 (0)	2 (100)
NA	10 (16.7)	0 (0)	0 (0)	0 (0)	2 (20.0)	0 (0)	3 (21.4)	0 (0)	1 (12.5)	2 (20.0)	2 (100)	0 (0)

MSM, men who have sex with men; Hetero, heterosexuals; MTC, mother-to-child; LAg-Avidity EIA, limiting-antigen avidity enzyme immunoassay; Recent, recent infection; LT, long-term infection; and NA, data not available.

Also, the other seven URFs_0107 clusters containing two to four individuals were observed in male patients (Table 2). Three clusters (I, III, and XI) contained both homosexual and heterosexual transmission, among which cluster I (*n* = 4) patients were all recently infected, while clusters III and XI were chronic HIV-1 infections. Four clusters (II, V, VII, and X) were transmitted by homosexual contact only, and recent HIV-1 infections were detected in cluster II, V, and VII.

### Potential Recombination Hotspots in the *pol* Region

**Figure 2:**
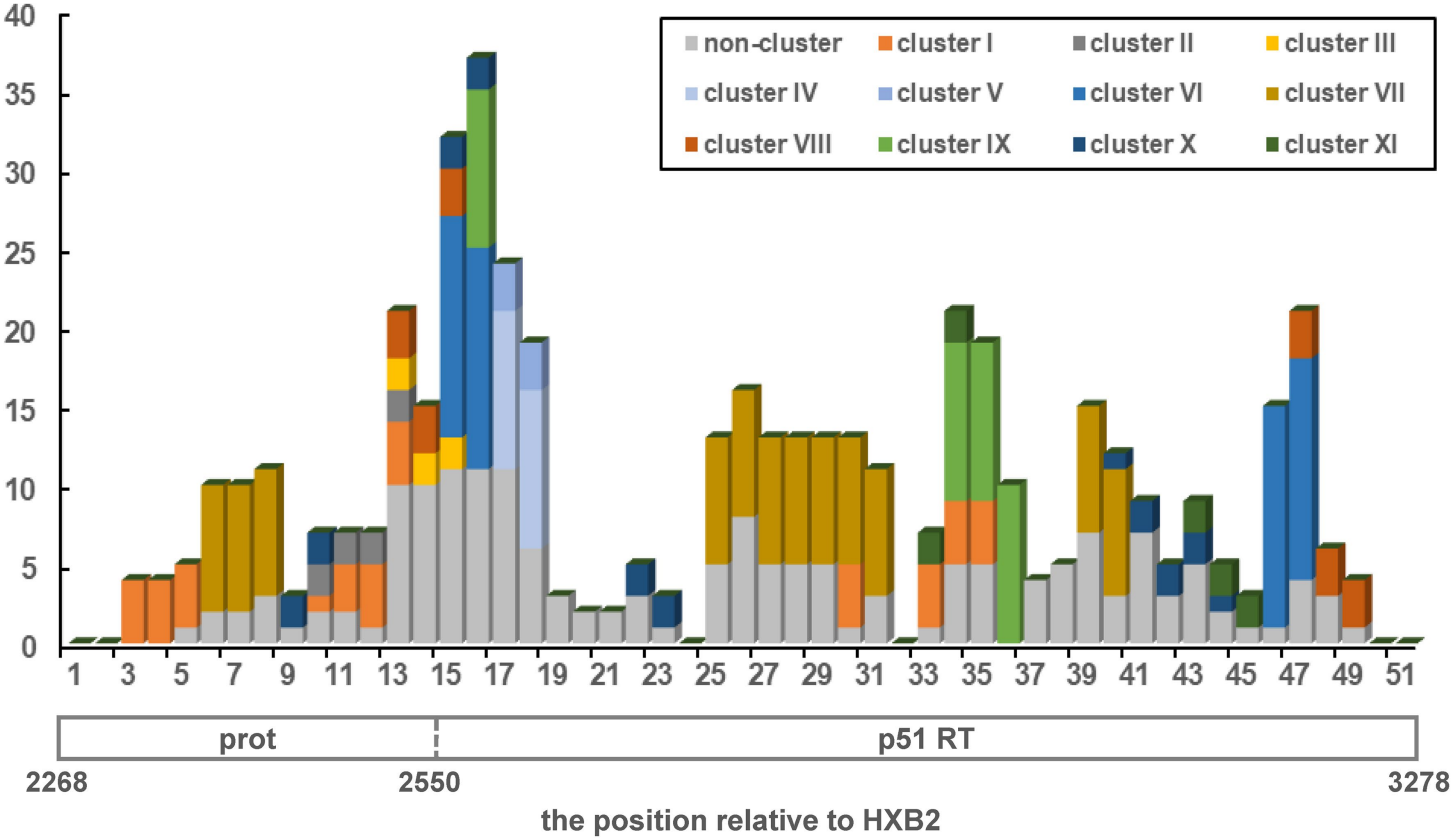
The distribution and frequency of breakpoints across pol region in HIV-1 URFs_0107 strains (HXB2: 2268-3278). The *x*-axis shown the position relative to the protease (prot) and reverse transcriptase (RT), while the *y*-axis indicated the breakpoint frequency. The 1.1-kb *pol* region was divided into 51 windows using 20 non-overlapping nucleotide steps. Eleven transmission clusters were color-coded, and the non-clustered sequences were uniformly represented in gray.

Subsequently, we conducted a simple recombination breakpoint scanning on the 110 URFs consisting of CRF01_AE and CRF07_BC. After excluding five sequences with ambiguous breakpoints, there were 191 breakpoint positions recorded among the 105 sequences, with some sequences having more than one breakpoint. These positions were plotted as a frequency plot of breakpoints in Figure 2. Overall, the breakpoints of URFs_0107 strains were widely distributed, covering almost the 1.1 kb *pol* genomes. One clear recombination peak can be observed with a position of 2,508–2,627 relative to HXB2 genome, near the junction of protease and reverse transcriptase genomes. There were 83.3% (50/60) of clustered and 24.4% (11/45) of non-clustered URFs observed breakpoints at this hotspot region, respectively. Moreover, we also found that some clustered URFs_0107 have similar breakpoints in other regions, such as cluster I and VII at 2848–2867, and the cluster I, IX, and XI at 2908–2967 (position relative to HXB2).

### Variety of Recombination Patterns and Distinct Parental CRF01_AE Origins in URFs_0107

**Figure 3:**
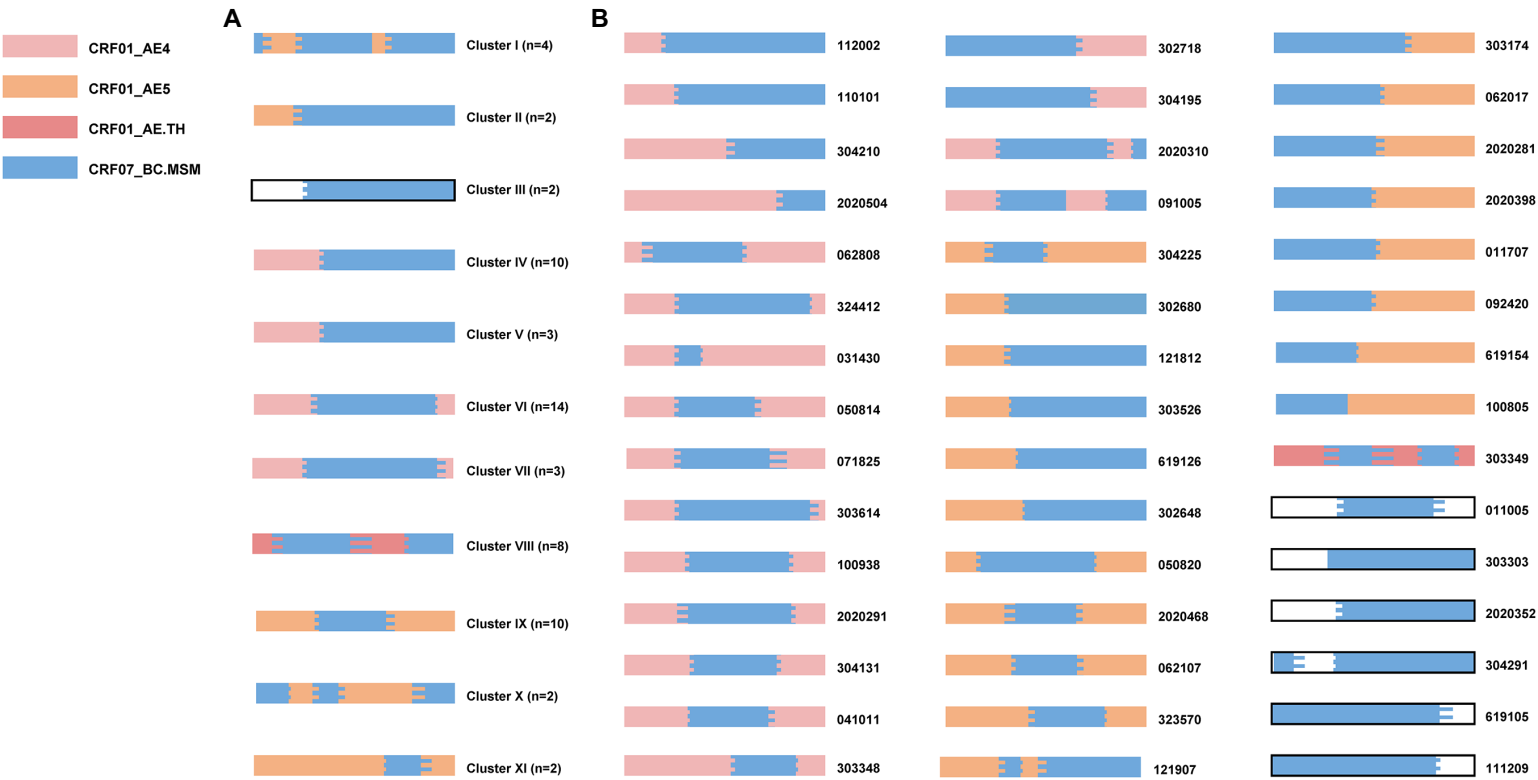
The recombination patterns and parental origin of partial *pol* region in URFs_0107 clustered sequences **(A)** and non-clustered sequences **(B)**. The recombination structures were confirmed by RIP, JPHMM, and Simplot software (v 3.5.1). The parent origins of the fragments were represented by different colors, among which blank represented that the definite lineages of CRF01_AE were not identified due to the short sequence fragments or ambiguity bases. The sample codes were indicated on the right side of the mosaic map. The mosaic genetic map was generated by the Recombinant HIV-1 Drawing online tool (www.hiv.lanl.gov/content/sequence/DRAW_CRF/recom_mapper.html).

To further dissect the HIV-1 URFs_0107 epidemics, we performed a detailed analysis of recombination patterns and parental origin in 1.1 kb *pol* region (Figure 3; Supplementary Figure S2), and the 5′ half-genome sequences of some specimens were used to supplement the evidence (Supplementary Figure S3). The parental origin of the recombinant CRF01_AE fragments was multiple. Of the 60 clustered URFs_0107 strains, the CRF01_AE fragments of clusters IV-VII originated from CRF01_AE lineage 4 (50%, 30/60), the clusters I-II and IX-XI derived from CRF01_AE lineage 5 (33.3%, 20/60), as well as the parental CRF01_AE of cluster VIII originated from CRF01_AE lineage Thailand (13.3%, 8/60). For the 45 non-clustered URFs_0107 strains, the proportion of parentals derived from CRF01_AE lineage 4, lineage 5, and lineage Thailand was 42.2% (19/45), 42.2% (19/45), and 2.2% (1/45), respectively. In addition, eight cases whose origin is not clear due to the shorter CRF01_AE fragments or ambiguity bases. In contrast, the CRF07_BC fragments of all URF_0107 strains were derived from CRF07_BC lineage predominant in MSM, which were significantly phylogenetically distinct from CRF07_BC strains prevalent among injecting drug users (Zhang et al., 2017).

**Figure 4:**
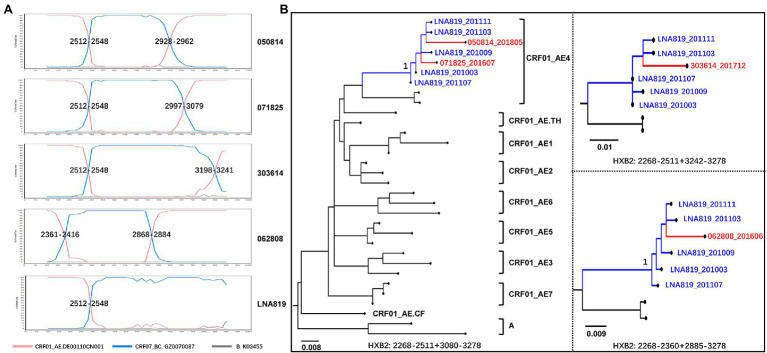
The recombination structure **(A)** and evolutionary relationship **(B)** of four URFs_0107 strains and LNA819. **(A)** Recombination analysis of four non-clustered URFs_0107 in the present study and LNA819 related strain (MT857722) by SimPlot (v3.5.1). **(B)** The subregion trees of CRF01_AE homologous region of recombinants were constructed by IQ-TREE under the GTR + I + G model with 1,000 replications. The red branches represented the URFs_0107 in this study, and the blue branches represented the continuous-time CRF01_AE strains in LNA819. The reference sequences were downloaded from the HIV-1 database, including seven lineages of CRF01_AE in China, lineage Thailand, and Africa, as well as subtype A. Only the key bootstrap values are shown.

The recombination structures of URFs_0107 were various. Among the URFs_0107 originals from CRF01_AE lineage 4, the common recombination structure was 01_AE/07_BC/01_AE (57.1%, 28/49), followed by 01_AE/07_BC (34.7%, 17/49), 01_AE/07_BC/01_AE/07_BC (4.1%, 2/49), and 07_BC/01_AE (4.1%, 2/49). And, five patterns were observed in the URFs_0107 originals from CRF01_AE lineage 5, including of 01_AE/07_BC/01_AE (43.6%, 17/39), 01_AE/07_BC (17.9%, 7/39), 07_BC/01_AE (20.5%, 8/39), 07_BC/01_AE/07_BC/01_AE/07_BC (15.4%, 6/39), and 01_AE/07_BC/01_AE/07_BC (2.6%, 1/39). In addition, only one recombination pattern of 01_AE/07_BC/01_AE/07_BC/01_AE was observed for URFs_0107 from CRF01_AE lineage Thailand. Of note, four non-clustered URF_0107 strains were found to have the CRF01_AE lineage 4 fragments closely related to strains in superinfection individual LNA819 under the subregion analysis (Figure 4). Our previous study showed that LNA819 was a CRF01_AE4/CRF07_BC superinfection with extremely active high-risk behavior, and a variety of distinct recombinants produced in LNA819 have been detected in the other five infected patients (Gao et al., 2021). The recombination structure of four URFs_0107 was 01_AE/07_BC/01_AE, and three of them had identical breakpoint at the position of 2,512–2,548, which also be seen in LNA819 (Figure 4A). The topologies of the subregion tree showed that the CRF01_AE fragments from four URFs_0107 were rooted in the pure CRF01_AE strains from LNA819 (five series of sampling points between 2010 and 2011), formed a monophyletic cluster with bootstraps value 1 (Figure 4B). Taken together, the similar breakpoints and high homologous parental strains suggested a close evolutionary relationship among four URFs_0107 strains and LNA819.

## Discussion

We performed a detailed characterization of the molecular epidemiology of URFs in Shenyang, northeast China from 2016 to 2020. The recombination patterns of URFs were complicated and dominated by CRF01_AE/CRF07_BC. The parentals of URFs_0107 were derived from the strains circulating among MSM, and the high level of recent infections indicated that URFs_0107 is continually emerging and spreading. Our data suggested the importance of continuous and accurate molecular epidemiology surveillance of recombinants, as well as provided an application method to molecular tracing of HIV-1 URFs for other regions in China where predominantly transmit HIV-1 among MSM population.

First, this study confirmed CRF01_AE/CRF07_BC was the most prevalent HIV-1 URFs in Shenyang over the last 5 years. Multiple subtypes have been reported from the beginning of the HIV epidemic in Shenyang. In the early 2000s, HIV-1 transmission was mainly through blood donation/transfusion and heterosexual contact in Shenyang, and the subtype B/B′ and CRF01_AE were the common prevalence strains (Han et al., 2001, 2010). Thus, the CRF01_AE/B was the dominant recombinants during the same period (Han et al., 2010, 2013b). However, since the year 2006, the prevalence of HIV-1 among MSM population has rapidly expanded, by far, the homosexual has transformed into the predominant risk-group of HIV-1 transmission with dominated circulating subtype CRF01_AE and CRF07_BC (An et al., 2012; Han et al., 2013c). Based on our recent analysis of the *pol* gene, the prevalence of CRF01_AE and subtype B gradually declined in the city during 2008–2016, while a significant increase of subtype CRF07_BC and other CRFs/URFs was reported in Shenyang (Liu et al., 2020). The trend with a higher proportion of URFs indicated the increasing viral complexity of HIV-1 epidemic in Shenyang and the similar situation would also be present in other regions of the national.

Second, the URFs_0107 identified in this study had distinct origin of CRF01_AE fragments, including the CRF01_AE lineage 4 and lineage 5, which have been reported to be widespread among Chinese MSM (Wang et al., 2017), as well as the lineage from Thailand, which is distinct from the seven lineages of CRF01_AE commonly circulating in China (Feng et al., 2013). In fact, the prevalence of CRF01_AE lineage 5 (75.3%) is much higher than lineage 4 (21.2%) in Shenyang, compared with other cities (Han et al., 2013c; An et al., 2021). However, our results showed that the number of URFs_0107 whose parents were CRF01_AE lineage 4 (*n* = 49) was higher than that of lineage 5 (*n* = 39). This is an indication that the URFs_0107 with CRF01_AE4 as parent might have been introduced into the Shenyang from other provinces, or patients infected with the CRF01_AE4/CRF07_BC recombinants have more active risk behaviors, resulting in widespread local transmission. Additionally, four large potential transmission clusters were found (IV, VI, VIII, and IX), indicating that these recombinants are already obtaining the ability to spread in this region (Table 2). It has been reported that recombinant forms can display enhanced replicative fitness compared with parental strains (Njai et al., 2006; Lau et al., 2010) and can have increased pathogenicity and virulence (Fischetti et al., 2004). Meanwhile, cross-transmission events between MSM and heterosexual were identified in six transmission clusters (I, III, IV, VI, VIII, IX, and XI) which may increase the risk of further expansion of the URFs_0107 strains to general population in Shenyang (Table 2). And, there is a significant imbalance between male (*n* = 11) and female (*n* = 1) patients infected through heterosexual transmission, indicating the possibility of male patients concealing their true sexual orientation.

Third, the determination and intervention of the transmission sources are of great significance for identifying how strains continue to spread and how to avoid new infections effectively. Phylogenetic and distance-based analysis are common strategies to determine the possible transmission networks (Trask et al., 2002; Smith et al., 2009; Little et al., 2014). However, when many recombinants are put together for analysis, they tend to affect the topology of the phylogenetic tree due to the distribution of recombination breakpoints. Recombinants with similar breakpoints are more likely to cluster together in the evolutionary tree, but their parental origin may be different (He et al., 2020). Meanwhile, multiple infections can produce various recombinants, and the strains with different backbone or breakpoints from the same source on the evolutionary tree may not cluster. This phenomenon also appeared in the patient of this study. The parental of CRF01_AE fragment within the four non-clustered strains with different recombination breakpoints was traced back to LNA819, a superinfected person previously identified. The case was diagnosed with HIV-1 CRF01_AE infection in March 2010, re-infected by CRF07_BC in December 2010, and started antiretroviral therapy in 2014 (Luan et al., 2017; Gao et al., 2021). Our analysis showed that four URFs_0107 identified in 2016–2018 were closely related to the evolution of the pure CRF01_AE strains in LNA819, suggesting the possibility of indirect transmission. The patient LNA819 could generate and transmit various recombinants during the treatment-naïve period, or transmit pure CRF_01AE strains to recipients, who could be superinfected with another CRF07_BC strain to develop a series of new URFs_0107 for further spread. It reminded us that the transmission network associated with LNA819 was not effectively interfered, and the related strains could spread lasted up for 8 years. Thus, it is strongly necessary to trace the source of the homologous recombinant fragments for inferring the potential transmission chain, control the early stages of infected patients in timely, monitor the uninfected persons who have contact with the infected, and guide the implementation of Pre-Exposure Prophylaxis (PrEP).

Finally, we found that 58.1% (61/105) of URFs_0107 have similar breakpoints in *pol* region around the position of the junction of protease and reverse transcriptase (Figure 2), indicating that this region might be the hotspots of recombination. There may be several reasons for similar breakpoints in recombinants. For example, the similar recombination breakpoints of CRF07_BC and CRF08_BC were attributed to the back-cross between common URF_BC and subtype C in Yunnan, China (McClutchan et al., 2002); CRF74_01B shared four and two breakpoints with CRF33_01B and CRF53_01B, respectively, because CRF74_01B may be a direct descendant of these two CRFs (Cheong et al., 2015). In addition to the above-mentioned recombinants that contain evolutionary relationships, another reason for the formation of common breakpoints is due to the sequence similarity, hairpin structure of genomic RNA, fragile and pause sites, and high-pairing probability (Galetto et al., 2004; Galetto and Negroni, 2005; Delviks-Frankenberry et al., 2011; Jia et al., 2016). The other studies also found hotspots at the genomic junction of protease and reverse transcriptase, which was consistent with our findings (Galli et al., 2008; Smyth et al., 2014).

The major limitations of this study are that (1) only by 1.1-kb *pol* region sequence may underestimate the prevalence of recombination. However, using the pol sequences from the routine drug-resistant test will contribute to the real-time monitor the change of HIV recombinants, which is the most focus in this study; (2) we tried to obtain as many as 5′ half-genome sequences as possible, but considering time and availability of resources, only 14 patients 5’half-genome sequences were amplified to support or verify the recombination analysis of the pol region. In the future, we need to further obtain more 5′-half-genome or the nearly full-length genome sequences of patients to trace the source of the recombinants, clarify the evolutionary relationship; and (3) due to the protection of personal privacy, we do not have enough epidemiological data to determine whether there is a real direct/indirect transmission relationship among clustered URFs.

In conclusion, our study provides molecular evidence that the unique recombination patterns lead to a complex HIV-1 epidemic in Shenyang. In recent years, the transmission of URF_0107 strains among MSM in Shenyang has increased, some of which have caused more widespread transmission. Our study highlights the importance of continued and accurate molecular surveillance to increase our understanding of the evolving HIV-1 URFs epidemic. And tracing the source of recombinants is necessary, which helps provide specific interventions to the most relevant high-risk population.

## Data Availability Statement

The original contributions presented in the study are included in the article/Supplementary Material, further inquiries can be directed to the corresponding authors.

## Ethics Statement

The studies involving human participants were reviewed and approved by the First Affiliated Hospital of China Medical University. The patients/participants provided their written informed consent to participate in this study.

## Author Contributions

HS and XH conceived and designed the study. WS and QL collected the specimen information. SH, GG, LW, YG, and WT performed experimental work. MA and BZ performed data collection. SH performed data analysis and wrote the first draft. All authors contributed to the article and approved the submitted version.

## Funding

This work was funded by the National Natural Science Foundation of China (81871637 and 82072272), CAMS Innovation Fund for Medical Sciences (2019-I2M-027), and Science and Technology Project of Shenyang (19-104-4-026).

## Conflict of Interest

The authors declare that the research was conducted in the absence of any commercial or financial relationships that could be construed as a potential conflict of interest.

## Publisher’s Note

All claims expressed in this article are solely those of the authors and do not necessarily represent those of their affiliated organizations, or those of the publisher, the editors and the reviewers. Any product that may be evaluated in this article, or claim that may be made by its manufacturer, is not guaranteed or endorsed by the publisher.

## Footnotes

^1^ https://www.hiv.lanl.gov

^2^ https://bioedit.software.informer.com/7.0/

^3^ https://www.hiv.lanl.gov/content/sequence/DRAW_CRF/recom_mapper.html
