# Supplementary material for: Multiple CRF01_AE/CRF07_BC Recombinants Enhanced the HIV-1 Epidemic Complexity Among MSM in Shenyang City, Northeast China
Source: Front Microbiol. 2022 May 12;13:855049. doi: 10.3389/fmicb.2022.855049 (PMC9133626; doi:10.3389/fmicb.2022.855049)
Supplement: Supplementary file 2 [file Table_2.DOCX]

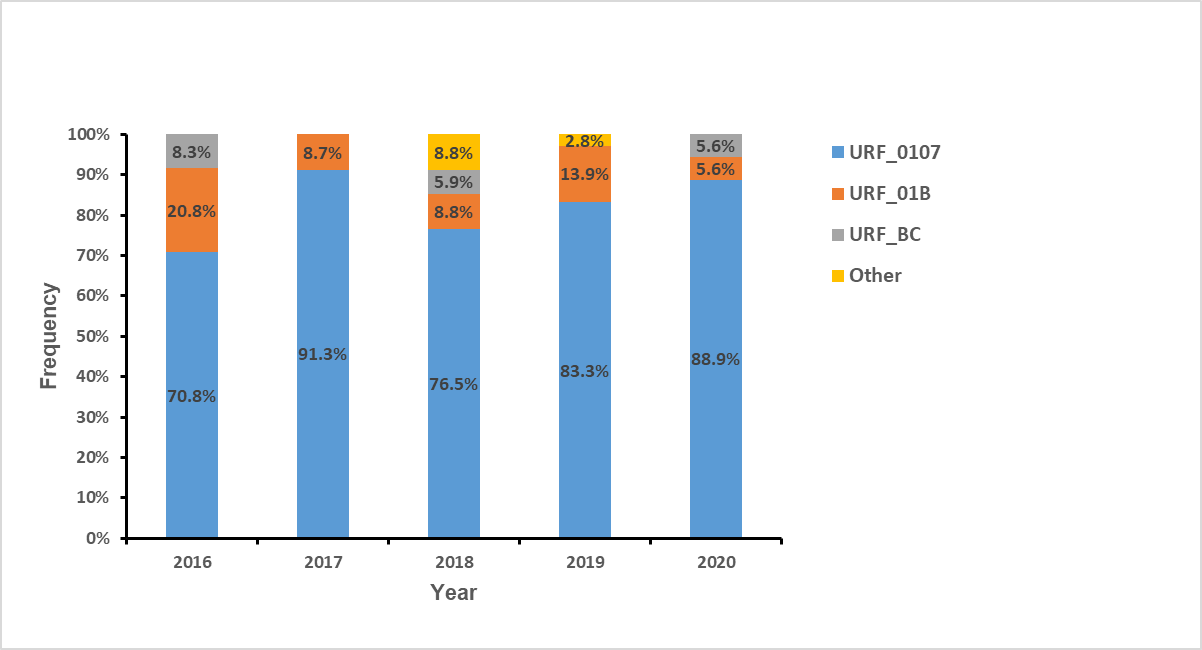


Supplementary Figure S1. Composition of newly diagnosed HIV-1 URFs infected cases from 2016 to 2020. Blue, orange, gray, and yellow columns represented the URF_0107, URF_01B, URF_BC, and other recombinants, respectively.


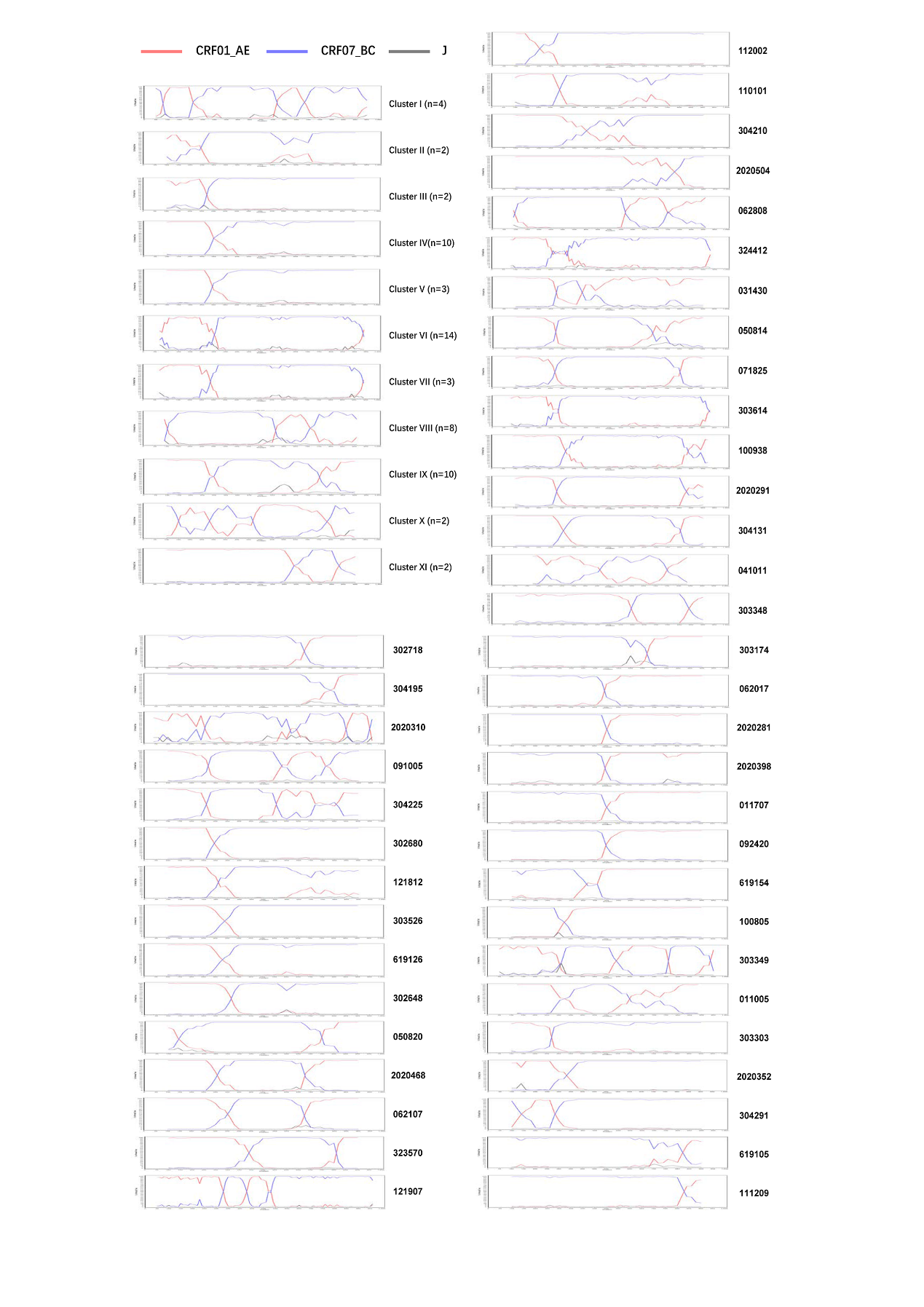


Supplementary Figure S2. Bootscan analysis of pol region (HXB2:2268-3278) of the URFs_0107 strains by Simplot (version 3.5.1). CRF01_AE and CRF07_BC were used as parental strains and subtype J was used as an outgroup. SimPlot parameter selection criteria were 200 bps window size and 20 bps step size.


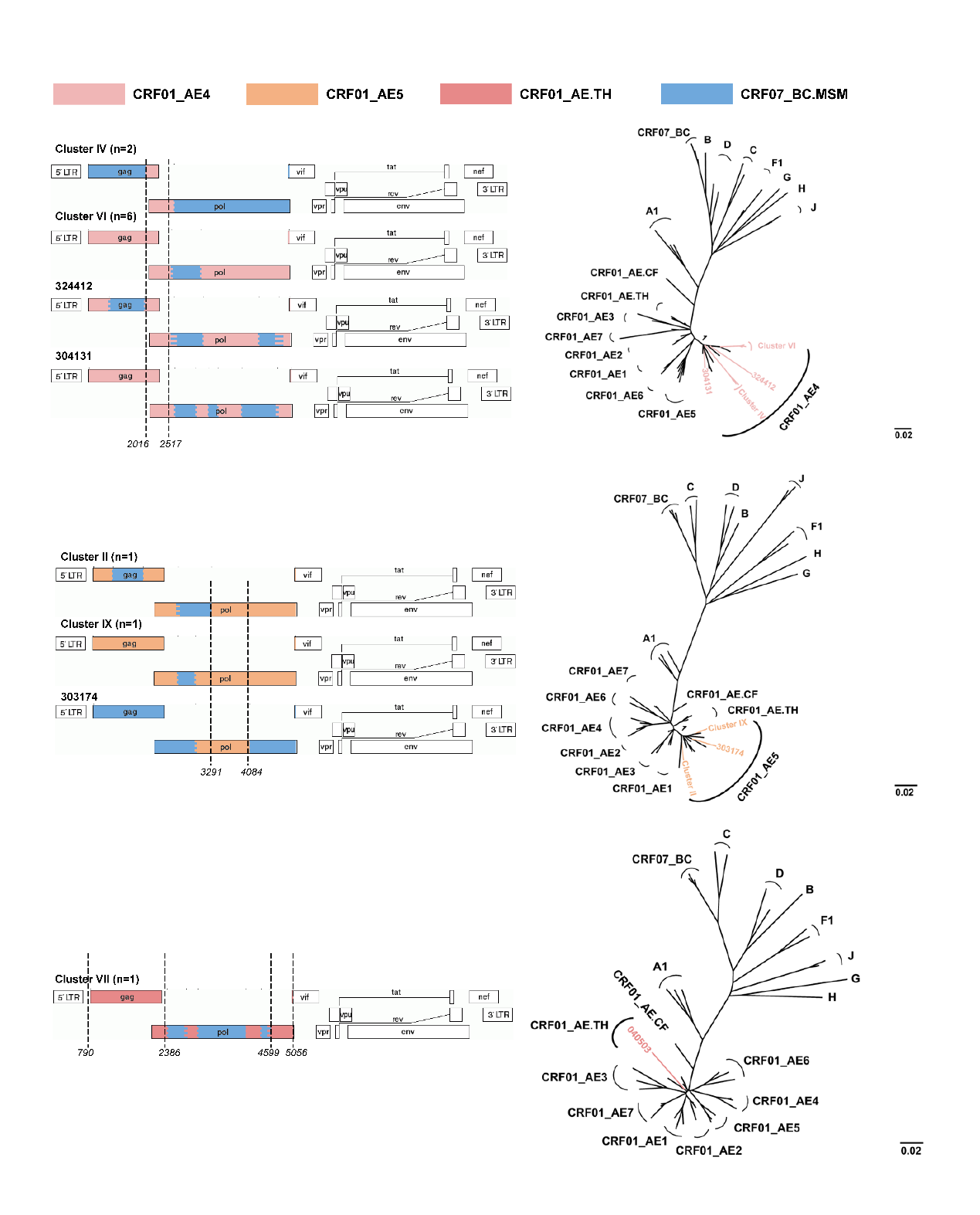


Supplementary Figure S3. The recombination patterns and parental origin of 5’-half genome sequences in HIV-1 URFs_0107 strains. The recombination structures were confirmed by RIP, JPHMM, and Simplot software (v 3.5.1). The origins of the fragments were represented by different colors, and the mosaic genetic maps were generated by the Recombinant HIV-1 Drawing online tool (www.hiv.lanl.gov/content/sequence/DRAW_CRF/recom_mapper.html). The ML trees of CRF01_AE homologous fragments were constructed under the GTR+I+G model with 1000 replications by IQ-TREE. The reference sequences included seven lineages of CRF01_AE in China, CRF07_BC, and the pure genotypes of group M.
